# Double-Network Hydrogels via Hybrid Strategies: Potential in Large-Scale Manufacturing for Colorimetric Indicator

**DOI:** 10.3390/gels11090697

**Published:** 2025-09-02

**Authors:** Ningli An, Jiwen Liu, Wentao Zhou, Qing He, Jianan Li, Yali Xiong

**Affiliations:** Faculty of Printing, Packaging Engineering and Digital Media Technology, Xi’an University of Technology, Xi’an 710054, China; 2230821160@stu.xaut.edu.cn (J.L.); xutwentao@163.com (W.Z.); 2230820060@stu.xaut.edu.cn (Q.H.); 2240820063@stu.xaut.edu.cn (J.L.); 18886369496@163.com (Y.X.)

**Keywords:** double-network, colorimetric, sodium alginate, agar, gelatin, PVA

## Abstract

Biological hydrogels are widely available in terms of raw material sources and can be processed and molded using relatively simple techniques. Hydrogels can offer abundant three-dimensional, water-containing channels that facilitate the reaction between gases and dye, making them the preferred choice for the solid support layer in colorimetric indicators. However, biomass hydrogels exhibit inferior mechanical properties, making them unsuitable for large-scale manufacturing processes. In this study, four dual-network composite hydrogels Agar/Gelatin, Sodium Alginate/Agar, Sodium Alginate/Poly (vinyl alcohol), Sodium Alginate/Gelatin (AG/Gel, SA/AG, SA/PVA and SA/Gel) prepared through hybrid strategies. Furthermore, the influence of the dual-network structure on the mechanical properties and ammonia response was systematically investigated, using microscopy and Fourier transform infrared spectroscopy (FTIR) characterization method. The experimental results demonstrate that the incorporation of SA into original hydrogel matrices can significantly enhance both the mechanical and ammonia response performance due to the secondary topological network structure. The interpenetrating double network structure was effectively regulated through the calcium ion cross-linking process. The color difference threshold of SA/PVA’s response to ammonia gas is 10, it holds promise for rapid detection applications. The SA/Gel composite hydrogel exhibits excellent mechanical robustness and toughness. The tensile strength of the SA/Gel sample is 11 times that of a single gel, and the toughness is 80 times greater, suggesting its suitability for large-scale manufacturing of colorimetric indicator.

## 1. Introduction

Hydrogels are a type of hydrophilic polymer network system that can be formed through intermolecular interactions (such as hydrogen bonds, electrostatic interactions, hydrophobic interactions, etc.) or covalent cross-linking. It has a rich three-dimensional porous structure [1,2]. The structural characteristics of hydrogels endow them with unique physical and chemical properties, enabling them to respond to external environmental stimuli. Therefore, hydrogels have broad application prospects in fields such as flexible electronics [3,4], biomedicine [5], smart packaging [6,7] and other fields. Bio-based hydrogels are derived from natural materials and have advantages such as renewability, biocompatibility and biodegradability. Common materials include alginate [8], gelatin [9], agar [10,11], chitosan [12], starch [13], and cellulose [14].

Alginate is an anionic natural polysaccharide salt extracted from alginic acid. Its main chain is rich in hydroxyl and carboxyl groups and is easy to modify. These provide multiple reaction sites and favorable conditions for the functionalization of sodium alginate [15,16]. Gelatin is a derivative of collagen. Due to its ability to mimic extracellular matrices, it is widely used as a functional material in daily life and research. As a polyampholyte, it forms ion pairs between its charged network and counterions. It can regulate the electrostatic interaction within its molecular chain. Therefore, the mechanical properties of gelatin are affected by ions [17,18].

Agar (AG), a polysaccharide extracted from seaweed, has attracted attention in recent years due to its excellent transparency and biocompatibility. The viscosity of AG solution shows a temperature dependence. When the AG hot solution is cooled below 60 °C, the AG molecular chains initially form disordered semi-rigid helical coils. When the temperature is below 40 °C, the molecular chains undergo ordered rearrangement and eventually form a three-dimensional network structure. This distinctive characteristic ensures its high compatibility with a wide range of Macro manufacturing technology and micro-nano manufacturing technology [19,20]. Poly (vinyl alcohol) (PVA) is a water-soluble synthetic polymer produced through polyvinyl acetate hydrolysis. The crosslinking degree of PVA hydrogels can be increased through freeze-thaw cycles, which eliminates the need for chemical crosslinking agents. During this process, PVA chains gradually crystallize, leading to the formation of a stable three-dimensional network structure [21].

The aforementioned materials have exhibited distinct advantages across a range of application domains. AG is recognized as one of the most promising biomass-derived hydrogels with potential applications in 3D printing, Imprinting, and other advanced manufacturing technologies [22,23]. Gelatin exhibits excellent mechanical toughness and is widely regarded as a preferred material for flexible wearable devices. Sodium alginate (SA) demonstrates pH sensitivity, making it a suitable choice for pH sensor applications [24,25,26]. Polyvinyl alcohol (PVA) can achieve superior mechanical properties through repeated freeze-thaw cycles. By employing a physical mixing approach, the distinctive properties of the constituent components can be synergistically integrated to design advanced materials tailored for specific applications. The synthesis of such hydrogels typically avoids the use of toxic crosslinking agents [27].

Colorimetric indicator are a type of simple, efficient, and low-cost gas sensors that change color in response to external stimuli, thereby indicating changes in food quality [28]. They can trace food sources, monitor freshness in real time, and provide timely information on quality changes, ultimately enhancing product safety and quality by offering early warnings of potential issues [29,30]. Generally, a colorimetric indicator consists of two parts: a solid support layer and an indicator combined with it [31]. Hydrogels, as natural biological materials, are cost-effective, biocompatible, and environmentally friendly, making them suitable candidates for solid support layers [32,33].

However, biomass hydrogels produced through conventional methods exhibit inferior mechanical properties [34,35,36], making them unsuitable for large-scale manufacturing processes such as roll-to-roll production. On the other hand, the performance of hydrogel-based colorimetric indicators is closely associated with the moisture retention capability of the solid support layer. Consequently, most studies of colorimetric indicators employ aerogel as the solid support layer. Our previous series of studies proposed an encapsulation layer structure, which effectively addressed the moisture retention issue in the hydrogel-based colorimetric indicators [31]. Obtaining a dual-network interpenetrating structure through physical blending can significantly improve the mechanical properties of hydrogels, thereby offering technical support for the large-scale production of hydrogel-based colorimetric indicators. The further development of the hydrogel network structure is a critical factor influencing the colorimetric performance.

**Figure 1:**
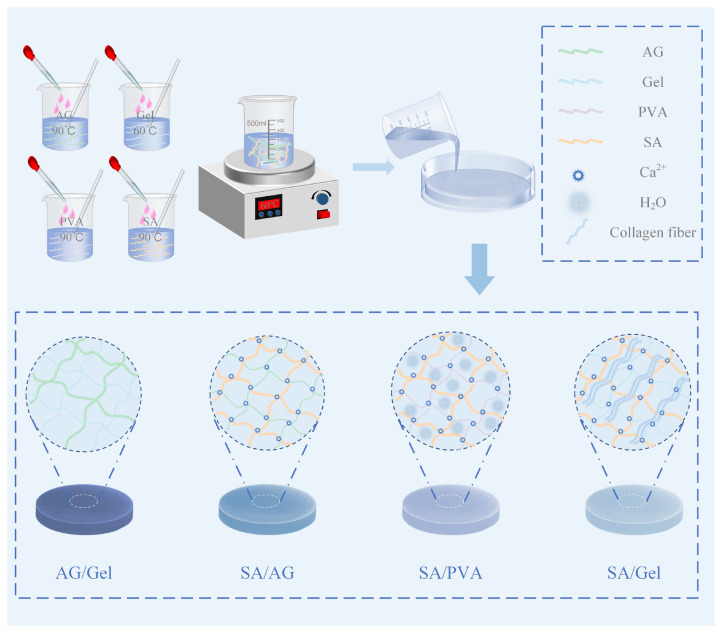
Preparation procedures and structural schematic of double-network hydrogels AG/Gel, SA/AG, SA/PVA, and SA/Gel.

In this study, four hydrogel materials—sodium alginate (SA), agarose (AG), polyvinyl alcohol (PVA), and gelatin (Gel)—were chosen as the structural components of a dual-network system due to their superior biocompatibility and promising potential for commercial applications. Four composite hydrogels featuring interpenetrating dual-network architectures were synthesized through a physical blending and crosslinking methods. Figure 1 presents the preparation procedures and structural schematics of the double-network hydrogels AG/Gel, SA/AG, SA/PVA, and SA/Gel. The mechanical properties and the colorimetric response resolution to ammonia gas of these hydrogels, fabricated through various compositional combination strategies, were systematically evaluated. The correlation between different dual-network structures and both the mechanical performance and the colorimetric response of the hydrogel composites was analyzed using surface and cross-sectional morphological characterization and Fourier transform infrared (FTIR) spectroscopy analysis. This research provides a foundational basis for the large-scale production of colorimetric sensing labels for food freshness monitoring and contributes to the expansion of potential applications for bio-based hydrogels.

## 2. Results and Discussion

### 2.1. Surface Morphology of Dual-Network Hydrogels

**Figure 2:**
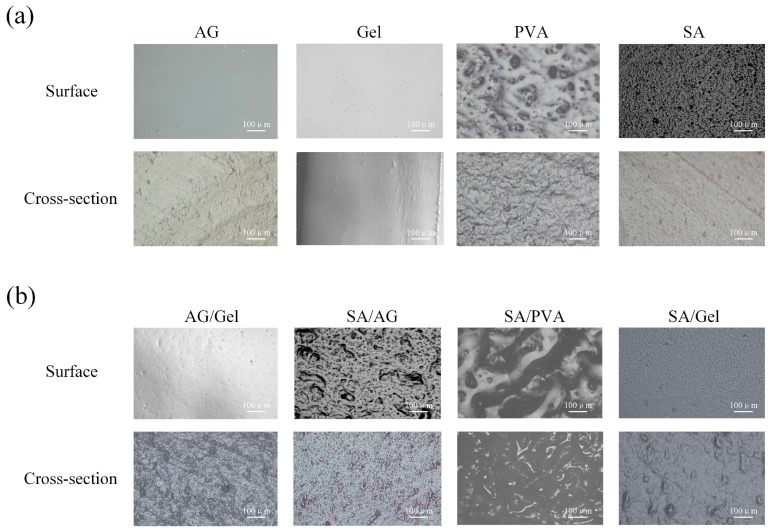
Optical microscopy images of hydrogel morphologies. (**a**) Surface and cross-sectional morphologies of single-network hydrogels: AG, Gel, PVA, and SA; (**b**) Surface and cross-sectional morphologies of double-network hydrogels: AG/Gel, SA/AG, SA/PVA, and SA/Gel.

The surface and cross-sectional morphologies of the AG, Gel, SA, and PVA hydrogel films were examined using two-dimensional optical microscopy. The corresponding images are shown in Figure 2a. The AG hydrogel film exhibits a dense and smooth surface morphology, whereas its cross-sectional structure reveals localized tearing and deformation. The gelatin sample displays a low surface pore density with a compact and smooth morphology, featuring a relatively uniform cross-sectional structure. The surface micrograph of the PVA hydrogel film reveals island-like interconnected morphologies, while its cross-sectional images show distinct interconnected fiber bundles, accompanied by free-water accumulation in localized concave or microporous regions. In contrast, the surface micrograph of the SA hydrogel film reveals dense granular protrusions, while the cross-sectional image exhibits oriented striations accompanied by free-water accumulation in the protruding regions.

The surface and cross-sectional morphologies of the dual-network hydrogels, formed by combining AG, Gel, SA, and PVA hydrogel components mixed in a 1:1 ratio, are presented in Figure 2b. Surface microscopy of the AG/Gel hydrogel film exhibited low-density distributed pores on its surface compared to pure AG, whereas its cross-section displayed uneven tearing with minor free water exudation. The surface microscopy of the SA/AG hydrogel film reveals an interconnected island-like structure formed on the surface, interspersed with medium-density concave pores. The cross-section of the SA/AG hydrogel film reveals uniformly distributed cross-linked protrusions, indicating effective interpenetrating network formation between the two components. Upon incorporation of SA into PVA, the resulting hydrogel exhibited surface and cross-sectional morphologies comparable to those of pure PVA, but with structural features magnified by tens of times in scale. Surface microscopy of the SA/Gel hydrogel, densely packed pores densely packed pores distributed across the interconnected island-like structures on the surface. Local regions of the cross-section displayed uniformly distributed plastic fracture necking. These findings indicate that that the collagen fiber bundle structure of gelatin facilitates an effective interpenetrating network within the SA matrix.

### 2.2. Mechanical Properties of Dual-Network Hydrogels

**Figure 3:**
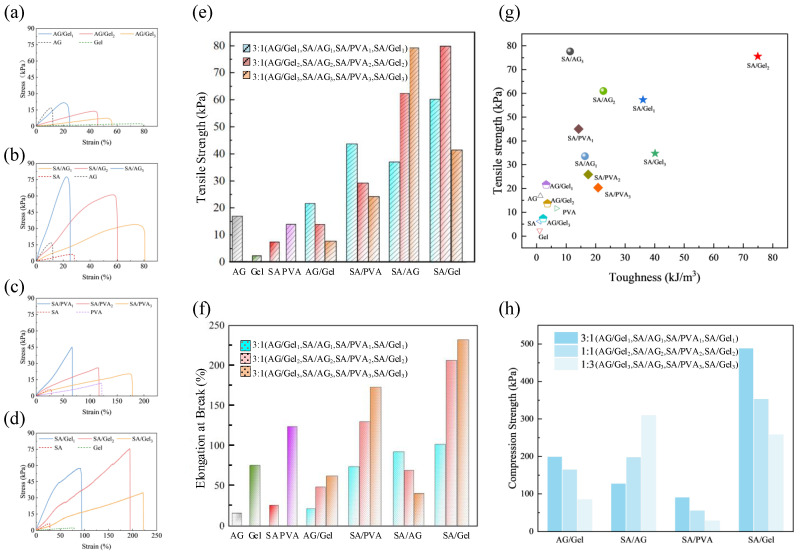
Mechanical properties of hydrogels. (**a**–**d**) Tensile stress-strain curves of double-network hydrogels AG/Gel, SA/AG, SA/PVA, and SA/Gel, along with their corresponding single-network hydrogels. (**e**) Tensile strength and (**f**) elongation at break of hydrogel materials. (**g**) Comparison of tensile strength and toughness of various hydrogels. (**h**) Compressive strength of double-network hydrogels.

Excellent mechanical properties are not only a fundamental requirement for applications in intelligent packaging, flexible wearable devices, and biomedical tissues, but also a essential prerequisite for large-scale manufacturing processes. The mechanical properties of the AG, Gel, SA and PVA hydrogels film, Gel/AG, SA/PVA, SA/AG and SA/Gel dual-network hydrogels films were assessed through tensile testing. The tensile stress-strain curves of composite hydrogels samples formed by four mixed strategies are presented in Figure 3a–d. Figure 3e compares the comparison results of tensile strength of all samples. Figure 3f compares the comparison results of elongation at break. Figure 3g presents the comparison results of strength and toughness. Figure 3h illustrates the comparison results of compressive strength.

The tensile strength of the Gel film (2.3 KPa) and the SA film (6.2 KPa) is lower than the PVA film (11.7 KPa) and AG film (16.7 KPa). Additionally, the elongation at break for AG (10.7%) and SA (27%) is lower than that of Gel film (77%) and PVA film (120.73%). Furthermore, toughness was quantified by calculating the area under the tensile stress-strain curve. Among the four materials evaluated, PVA demonstrates the best mechanical performance. This superior mechanical behavior can be attributed to the formation of a more effective crosslinking network during the freeze–thaw cycling process. Following this observation, a comprehensive investigation was carried out to assess the mechanical characteristics of four interpenetrating double-network hydrogels. The tensile testing results revealed that AG and Gel composite systems exhibit minimal physical crosslinking efficacy, with no notable enhancement in mechanical strength observed.

The experimental results indicate that the mechanical properties of SA/AG, SA/PVA, and SA/Gel hydrogels with a double-network structure are markedly enhanced in comparison to their single-network counterparts. This enhancement is presumably attributed to the formation of a rigid and compact second network by SA through calcium ion-induced crosslinking. Upon the establishment of this second rigid network, the interpenetrating polymer structure undergoes a topological reorganization. The resulting alternating rigid-flexible structural architecture demonstrates superior mechanical performance [37].

The tensile strength results, as shown in Figure 3e, reveal that SA/AG exhibits the most significant relative increase in tensile strength. This improvement can be attributed to the compressive effect exerted by the newly formed rigid network on the helical conformation of AG during gelation, which enhances both tensile and compressive strength. The mechanical strength of SA/AG increases with rising AG content. SA/PVA, benefiting from the inherent toughness of PVA, exhibits a notable increase in elongation at break. The second network in SA/PVA hydrogels is formed through ionic crosslinking between sodium alginate (SA) and multivalent calcium ions (${\mathrm{Ca}}^{2+}$). These ionic interactions act as sacrificial bonds that dissipate mechanical energy during deformation [38]. However, among the four hybrid systems evaluated, SA/PVA demonstrates the lowest compressive strength, likely due to its relatively high free water content, as observed in Figure 2b. The three SA/Gel hydrogel samples with different mixing ratios all exhibited considerable toughness and mechanical strength. The primary contributing factor is the interpenetrating structure formed between the rigid framework of SA and collagen fiber bundles present in Gel. The cross-sectional morphology of the SA/Gel hydrogel showed relatively uniform fiber bundle necking, as observed in Figure 2b. This dual-network topological structure is responsible for the robust mechanical properties of the SA/Gel hydrogels. Advances in the structural design and fabrication of interpenetrating networks enable the facile construction of high-strength, tough materials and high-performance biomimetic systems [39,40,41].

### 2.3. Structure of Dual-Network Hydrogels

**Figure 4:**
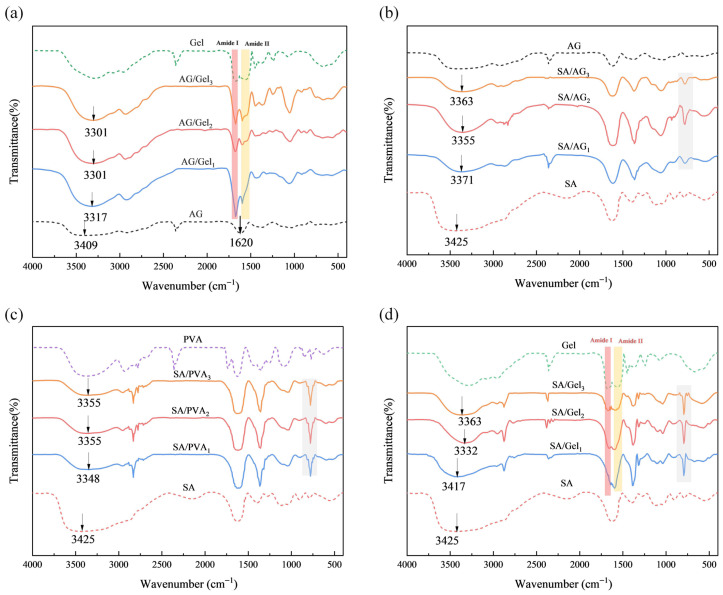
Fourier-transform infrared (FTIR) spectra of hydrogels. (**a**) FTIR spectra of double-network AG/Gel hydrogel and its corresponding single-network hydrogels; (**b**) SA/AG double-network and single-network hydrogels; (**c**) SA/PVA double-network and single-network hydrogels; (**d**) SA/Gel double-network and single-network hydrogels.

The formation of dual-network hydrogels with varying composition ratios (AG/Gel, SA/AG, SA/PVA, SA/Gel) and single hydrogels was characterized using FTIR spectroscopy, as shown in Figure 4. In the FTIR spectrum of SA, the bands at 3449 ${\mathrm{cm}}^{-1}$, 1615 ${\mathrm{cm}}^{-1}$, and 1440 ${\mathrm{cm}}^{-1}$ correspond to O-H stretching vibrations and the symmetric and asymmetric stretching vibrations of COO^−^ groups, respectively [42,43,44]. In the Gel spectrum, distinctive absorption bands appear at 1650 ${\mathrm{cm}}^{-1}$ (amide I, C=O stretching) and 1540 ${\mathrm{cm}}^{-1}$ (amide II, N-H bending) [45,46]. The AG spectrum shows prominent bands at 3400 ${\mathrm{cm}}^{-1}$ (O-H stretching) and 1070 ${\mathrm{cm}}^{-1}$ (C-O-C pyranose ring vibration) [47].

The spectra of SA/AG, SA/PVA, and SA/Gel dual-network hydrogels show a band shift near 3425 ${\mathrm{cm}}^{-1}$ toward lower wavenumbers. The data presented in Table 1 demonstrate redshift offsets upon incorporation of various hydrogel mixtures. This redshift is attributed to enhanced hydrogen bonding interactions arising from functional groups within the hydrogel matrix [48,49,50,51]. SA/AG and SA/Gel exhibited stronger peaks than SA/PVA, indicating a greater degree of molecular blending in the former two. These findings are consistent with mechanical testing results. The enhanced physical crosslinking observed in SA/Gel and SA/AG contributes to the formation of robust dual-network structures, thereby improving their mechanical properties. The characteristic peak of the 1:1 ratio sample among the three types of dual-network hydrogels is stronger than that of other ratios, and also exhibits a more pronounced redshift. These results indicate that the 1:1 ratio sample possess more uniform blending and demonstrate enhanced physical crosslinking effects.

Additionally, a distinct new peak near 840 ${\mathrm{cm}}^{-1}$ was observed in the FTIR spectra of the SA/PVA, SA/AG, and SA/Gel samples. This peak may be attributed to the formation of additional hydrogen bonds, potentially induced by conformational changes in glycosidic bonds. The data suggest that both SA/Gel and SA/PVA systems generate more hydrogen bonds within the dual-network structure, with the SA/Gel blend exhibiting a higher hydrogen bonding capacity among its functional groups compared to SA/PVA. This stronger network of intermolecular hydrogen bonds is directly correlated with the improved mechanical performance observed in the SA/Gel composites. The appearance of characteristic peaks at 840 ${\mathrm{cm}}^{-1}$ indicates that hydrogen bonding plays a significant role in supporting the formation of the secondary network structure of SA. The infrared spectra of gelatin exhibited characteristic amide I (1666 ${\mathrm{cm}}^{-1}$, C=O stretching) and amide II (1558 ${\mathrm{cm}}^{-1}$, N-H bending) bands, confirming its proteinaceous nature. In the AG/Gel system, the amide I band appeared at 1640 ${\mathrm{cm}}^{-1}$, overlapping with AG’s characteristic peak. Notably, the intensity of this composite peak correlated directly with the AG content [52]. For SA/Gel composites, the peak around 1600 ${\mathrm{cm}}^{-1}$ similarly overlapped with gelatin’s amide I band. Unlike AG/Gel, all SA/Gel samples exhibited a pronounced decrease in amide I band intensity [53,54]. Comparison of the amide band positions and intensities between AG/Gel and SA/Gel indicates strong intermolecular interactions between SA and Gel molecular chains. These interactions promote the formation of larger collagen fiber bundles within the interpenetrating topological network of the SA/Gel hydrogel. Collectively, the results demonstrate that SA and Gel chains form a robust interpenetrating network structure, which is consistent with the mechanical property tests—SA/Gel exhibits exceptional mechanical strength and toughness.

### 2.4. Color Response to Ammonia Vapor of Dual-Network Hydrogels

**Figure 5:**
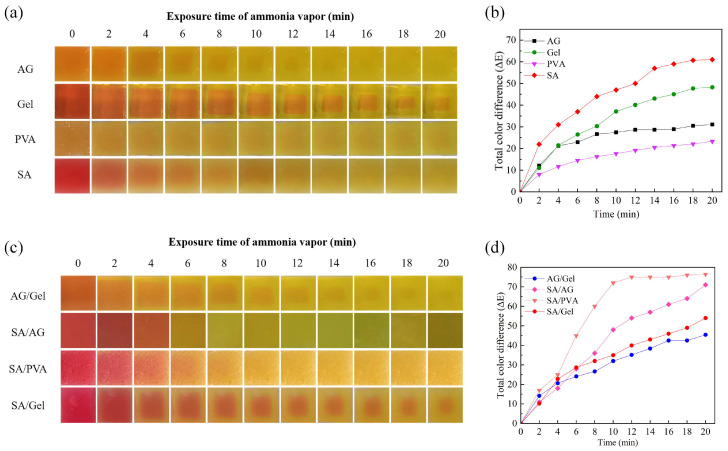
Ammonia responsiveness of hydrogels. (**a**) Colorimetric response of single-network hydrogels upon exposure to ammonia at various time intervals; (**b**) Temporal evolution of color difference (ΔE) of single-network hydrogels under ammonia exposure; (**c**) Colorimetric response of double-network hydrogels upon exposure to ammonia at various time intervals; (**d**) Temporal evolution of color difference (ΔE) of double-network hydrogels under ammonia exposure.

Figure 5a shows the significant color changes of the AG, Gel, SA, and PVA hydrogel samples within 20 min. Under controlled experimental conditions, including uniform solute concentrations and consistent indicator contents, differences in optical transparency resulted in distinct initial color appearances: AG hydrogel films presented as bright orange, Gel and SA hydrogel films exhibited a bright red hue, and PVA hydrogel films displayed a standard orange coloration. Following 20 min of exposure to an ammonia-rich environment, all four hydrogel samples transitioned from their initial colors to green. A comparative evaluation of pre- and post-exposure color states revealed that the SA and Gel samples indicated that the SA and Gel hydrogels underwent the most visually distinguishable color shifts. Throughout the exposure duration, each hydrogel sample exhibited unique spatiotemporal color distribution characteristics. PVA hydrogel films demonstrated uniform color distribution across the surface area. Both SA and AG samples displayed faint yet discernible chromatic halos, which gradually faded away by the end of the observation period. Gel hydrogels exhibited a faster chromatic transition at the periphery relative to the central region, with this spatial heterogeneity progressively diminishing over time.

The above results indicate that the inhomogeneous color distribution observed in the four single-network hydrogel samples is closely related to their water retention capacities. As shown in Figure 2a, PVA hydrogel exhibits a significantly stronger capacity to retain free water than the other three hydrogels, whereas Gel hydrogel demonstrates the weakest water retention performance. This significant disparity in water-retention behavior constitutes the principal factor underlying the non-uniform colorimetric responses exhibited by the four hydrogel samples. Figure 5c illustrates the color changes of AG/Gel, SA/AG, SA/PVA, and SA/Gel hydrogel samples over a 20-min period. The surfaces of the dual-network structures exhibit obvious color differences change over time. These composite hydrogels show uneven color distribution, characterized by gradual transitions between the central and peripheral regions. Among them, the SA/Gel interface maintains a comparatively sharp transitions boundary. The color change trend of AG/Gel samples is similar to that of AG, and the visual resolution is limited. The SA/PVA composite demonstrates reduced color uniformity compared to pure PVA. However, it achieves enhanced visual contrast, thereby improving colorimetric response performance.

Figure 5b depicts the color difference (ΔE values) of AG, Gel, SA, and PVA hydrogel samples over a 20-min period. While AG has reached the color difference threshold after 8 min, the remaining three samples demonstrated constantly color change throughout the observation period. The central regions of Gel and PVA followed close to linear change in their color difference variation curve. Notably, SA, as a pH-responsive hydrogel, manifested the most pronounced color difference variation among the four samples, owing to its pH-sensitive polymer network.

Figure 5d presents the ΔE values of AG/Gel, SA/AG, SA/PVA, and SA/Gel hydrogel systems. The AG/Gel and SA/Gel films displayed similar ΔE value. The variation curve of its color difference with time shows a linear relationship, attributed to Gel’s intrinsic colorimetric characteristic. Notably, the AG/Gel composite surpassed both of its single-network counterparts in color development performance and exhibited the highest linearity among all samples. Dual-network hydrogels containing SA showed relatively large ΔE values but also retained the nonlinear colorimetric behavior characteristic of SA. Although SA significantly enhanced the color response of PVA, it altered the inherent linear response behavior of PVA. The large-scale porous surface structure of SA/PVA, as shown in Figure 2b, endowed the hydrogel with excellent gas permeability and a high colorimetric response rate, enabling it to reach its maximum response threshold within 10 min—faster than the other samples. This finding suggests that the SA/PVA dual-network hydrogel holds strong potential for rapid detection applications. Table 2 shows the comparison of the color difference threshold results between the single gel sample and the double network structure sample. By comparison, it was found that the thresholds of the samples with a dual-network structure were all higher than those of the samples with a single-network structure.

## 3. Conclusions

Four distinct dual-network hydrogel architectures were fabricated through physical blending and ionic crosslinking methodologies. This investigation comprehensively evaluated the mechanical characteristics and ammonia vapor detection capabilities of the composite systems. Experimental data revealed that sodium alginate (SA) incorporation substantially enhanced both mechanical both the mechanical strength and colorimetric resolution of the resulting composite hydrogels. The tensile strength of the SA/Gel sample is 11 times that of a single gel, and the toughness is 80 times greater. The color difference threshold of SA/Gel and SA/AG is 20. Analysis of surface and cross-sectional morphologies, along with Fourier-transform infrared spectroscopy (FTIR) data, revealed that the secondary topological network structure formed during calcium ion crosslinking of SA, together with the intermolecular interactions among hydrogel chains, can effectively modulate the interpenetrating polymer network architecture of sodium alginate-based composite hydrogels. Therefore, future work may focus on optimizing process parameters to further enhance the mechanical and colorimetric performance of these composite hydrogels by precisely regulating the dual-network architecture.

Analysis of ammonia-responsive performance demonstrated that sodium alginate (SA) markedly improves the color difference resolution of polyvinyl alcohol (PVA), indicating that the SA/PVA composite hydrogel holds strong potential for applicability in rapid detection platforms. Furthermore, the SA/Gel composite hydrogel exhibits excellent mechanical robustness and toughness, suggesting its suitability for large-scale manufacturing of colorimetric indicator. While the SA/Gel composite achieves a high central color transition rate and favorable linear response, microscopic analysis revealed non-uniform color distribution. Notably, this uneven distribution presents distinct boundary features, offering distinct advantages for algorithmic feature extraction in computer vision systems. These collective properties position SA/Gel composite hydrogels as promising substrates for AI-integrated intelligent colorimetric arrays with scalable manufacturing capabilities.

## 4. Material and Methods

### 4.1. Materials

Sodium alginate (SA, viscosity 1.05–1.15 Pa·s) was obtained from Fuchen Chemical Reagents Co., Ltd. (Tianjin, China). Agarose (AG) was supplied by Beijing Solarbio Science & Technology Co., Ltd. (Beijing, China) Gelatin (Gel, $\tilde{2}$50 Bloom) was purchased from Aladdin Chemical Reagent (Shanghai, China). PVA (1799, analytically pure) and Methyl red (MR, 99%) were provided by Tianjin Kemiou Chemical Reagent Co., Ltd. (Tianjin, China). Glycerol (Gly) was sourced from Hunan Er-Kang Pharmaceutical Co., Ltd. (Changsha, China). Calcium chloride (CaCl_2_) was bought from Tianjin Tianli Chemical Reagent Co., Ltd. (Tianjin, China). Ammonia solution (25%) was obtained from Tianjin Fuyu Fine Chemical Co., Ltd. (Tianjin, China). Unless otherwise specified, all reagents and solvents were of analytical grade and used without further purification.

### 4.2. Preparation of AG, Gel, PVA, and SA Films

Methyl red (MR) powder (100 mg) was dissolved in 100 mL of absolute ethanol to prepare a 1 g per L MR stock solution. Then, 1.8 g of solute (AG, Gel, PVA, or SA) was added to 30 mL of deionized water and stirred under heating for 2 h; the heating temperature was set to 90 °C for AG and PVA, and 60 °C for SA and Gel. After complete dissolution, 0.4 mL of the 1 g per L MR solution and 0.4 mL of glycerol were added, and the mixture was further stirred at 60 °C and 400 rpm for 2 h to ensure thorough mixing. Subsequently, The film-forming solutions were then poured into Petri dishes using the solution casting method to obtain the films.

AG and Gel samples were crosslinked by cooling at room temperature. SA samples were crosslinked using a calcium ion crosslinking method by spraying ${\mathrm{CaCl}}_{2}$ solution (5 mL, 5% *w*/*v*) onto the surface and standing for 4 h. PVA films were subjected to three freeze-thaw cycles (frozen at −20 °C for 12 h, thawed at room temperature for 4 h, repeated three times) to induce physical crosslinking. The four film samples were designated as AG, Gel, PVA, and SA, respectively.

### 4.3. Preparation of Double-Network Hydrogel Films

Using the same temperature conditions as described in Section 2.2, AG, Gel, PVA, and SA were separately dissolved in deionized water with magnetic stirring. The polymer solutions were then blended at mass ratios of 3:1, 1:1, and 1:3 to prepare composite formulations of AG/Gel, SA/AG, SA/PVA, and SA/Gel. Each blended solution was adjusted to a total polymer concentration of 6% (*w*/*v*), and then supplemented with 0.4 mL of MR solution (1 g/L) and 0.4 mL of glycerol. The mixtures were stirred at 60 °C and 400 rpm for 2 h to ensure homogeneous mixing. Subsequently, the films were fabricated by the solution casting method. The AG/Gel samples were crosslinked by ambient cooling and were designated as AG/Gel1, AG/Gel2 and AG/Gel3 for the 3:1, 1:1, and 1:3 mass ratios, respectively. SA/AG samples were crosslinked using the same calcium ion crosslinking method described above by spraying CaCl_2_ solution (5 mL, 5% *w*/*v*) onto the film surface and allowing it to stand for 4 h. These were labeled SA/AG1, SA/AG2, and SA/AG3, respectively. SA/PVA samples were subjected to both freeze-thaw treatment (−20 °C for 12 h, then thawed at room temperature for 4 h, repeated three times) and calcium ion crosslinking, and named SA/PVA1, SA/PVA2, and SA/PVA3. Similarly, SA/Gel samples were treated with calcium ion crosslinking and designated as SA/Gel1, SA/Gel2, and SA/Gel3.

### 4.4. Characterization of Double-Network Hydrogel Films

#### 4.4.1. Surface Morphology

The surface and cross-sectional morphologies of single-network and double-network hydrogel films were observed using a metallographic microscope (GX71, Olympus, Tokyo, Japan) to evaluate surface uniformity and internal structural features.

#### 4.4.2. Mechanical Properties

Mechanical property testing of single-network and dual-network hydrogel films was conducted using a universal testing machine (UTM2103, Shenzhen, China) at room temperature, with each sample undergoing three replicate measurements. Tensile tests were performed at a speed of 50 mm/min. The films were cut into rectangular strips with a width of 10 mm, and their thickness was measured using a hand-held digital micrometer. Tensile strength (TS) and elongation at break (EB) were calculated using Equations (1) and (2), respectively:

(1) $$TS=\frac{F}{S}$$

(2) $$EB=\frac{\Delta l}{l_{0}}\times 100\%$$

where TS represents the tensile strength; *F*, the maximum load; *S*, the initial cross-sectional area of the film sample; EB, the elongation at break; Δ*l*, the extension of the films; *l*_0_, the initial test length of the films.

Compression tests were conducted at a compression rate of 5 mm/min. Hydrogel samples were prepared in the form of cylindrical specimens with a diameter of 20 mm and a height of 10 mm. The compressive strength (CS) was calculated using the following Equation (3):

(3) $$\sigma_{max}=\frac{F_{max}}{A_{0}}$$

where $\sigma_{max}$ is the maximum compressive strength, $F_{max}$ is the maximum load sustained before failure, and $A_{0}$ is the initial load-bearing area of the sample.

#### 4.4.3. Fourier Transform Infrared Spectroscopy (FTIR)

Fourier transform infrared spectroscopy was conducted to analyze the chemical structure of single-network and double-network hydrogel films. The samples were prepared by the potassium bromide (KBr) pellet method. Approximately 2 mg of lyophilized hydrogel was thoroughly mixed and ground with 100 mg of dry KBr powder, then compressed into pellets using a tablet press (Model FW-4). FT-IR spectra were recorded using a spectrometer (IRPrestige-21/FTIR-8400S, Shimadzu, Kyoto, Japan) in transmission mode. The analysis was performed within the wavenumber range of 4000–400 ${\mathrm{cm}}^{-1}$ with a resolution of 4 ${\mathrm{cm}}^{-1}$, and each sample was scanned 16 times. The obtained spectra were used to identify characteristic functional groups and to evaluate potential intermolecular interactions between polymer components.

#### 4.4.4. Ammonia Response of Hydrogel Films

The hydrogel samples were precisely cut into square blocks with dimensions of 10 mm × 10 mm and placed at the center of the upper cover of a circular sealed vessel. Then, 0.4 mL of ammonia solution with a concentration of 1.4% (*v*/*v*) is passed into the sealed circular container. The color change of the hydrogels was recorded in real time under ambient light using a digital camera (LYT900, Tokyo, Japan) positioned 170 mm directly above the sample surface. The CIE Lab color parameters ($L^{*}$, $a^{*}$, $b^{*}$) of specific regions were extracted using Adobe Photoshop 2021. Here, $L^{*}$ denotes the lightness index, with 0 indicating black and 100 indicating white; $a^{*}$ corresponds to the red-green axis, where positive values indicate red and negative values indicate green; $b^{*}$ corresponds to the yellow-blue axis, where positive values indicate yellow and negative values indicate blue. These color values were used to objectively evaluate the magnitude of chromatic variation and its visual perception. The total color difference (ΔE) was calculated using the following Equation (4):

(4) $$\Delta E=\sqrt{{\left(L^{*}-{L_{0}}^{*}\right)}^{2}+{\left(a^{*}-{a_{0}}^{*}\right)}^{2}+{\left(b^{*}-{b_{0}}^{*}\right)}^{2}}$$

where ${L_{0}}^{*}$, ${a_{0}}^{*}$ and ${b_{0}}^{*}$ are the initial color parameters of the films; and $L^{*}$, $a^{*}$ and $b^{*}$ are color values of the films at a specific ammonia level.

## Figures and Tables

**Table 1:** Peak shifts at 3330 ${\mathrm{cm}}^{-1}$ in the FTIR spectra of double-network hydrogels SA/AG, SA/PVA, and SA/Gel relative to the single-network SA hydrogel.

Sample	-OH Peak Position (cm^−1^)	Shift Relative to SA (cm^−1^)
SA	3425	–
SA/AG_1_	3371	54
SA/AG_2_	3355	70
SA/AG_3_	3363	62
SA/PVA_1_	3348	77
SA/PVA_2_	3355	70
SA/PVA_3_	3355	70
SA/Gel_1_	3417	8
SA/Gel_2_	3332	93
SA/Gel_3_	3363	62

**Table 2:** The color difference threshold (ΔE≤3).

Sample	AG	Gel	PVA	SA	AG/Gel	SA/AG	SA/PVA	SA/Gel
Time (min)	8	18	6	16	16	20	10	20

## Data Availability

The data presented in this study are available on request from the corresponding authors.

## Abbreviations

The following abbreviations are used in this manuscript:

AG	Agar
Gel	Gelatin
SA	Sodium Alginate
PVA	Poly (vinyl alcohol)
FTIR	Fourier transform infrared spectroscopy
Gly	Glycerol
${\mathrm{CaCl}}_{2}$	Calcium chloride
MR	Methyl red
